# Application of a Novel UPLC-MS/MS Method for Analysis of Rivaroxaban Concentrations in Dried Blood Spot and Plasma Samples Collected from Patients with Venous Thrombosis

**DOI:** 10.3390/molecules29174140

**Published:** 2024-08-31

**Authors:** Kornel Pawlak, Łukasz Kruszyna, Marta Miecznikowska, Marta Karaźniewicz-Łada

**Affiliations:** 1 Department of Physical Pharmacy & Pharmacokinetics, Poznan University of Medical Sciences, Rokietnicka 3, 60-806 Poznan, Poland; miecznikowska.marta.m@gmail.com (M.M.); mkaraz@ump.edu.pl (M.K.-Ł.); 2 Doctoral School, Poznan University of Medical Sciences, 60-812 Poznan, Poland; 3 Department of Vascular & Endovascular Surgery, Angiology and Phlebology, Poznan University of Medical Sciences, Dluga St 1/2., 61-848 Poznan, Poland; lukaszkruszyna@ump.edu.pl

**Keywords:** UPLC-MS/MS, rivaroxaban, plasma, DBS, venous thrombosis

## Abstract

Despite a higher safety profile compared to vitamin K antagonists, rivaroxaban therapy is still connected with multiple adverse effects, such as a high risk of bleeding. Thus, therapeutic drug monitoring (TDM) of rivaroxaban concentrations is suggested. An alternative to plasma samples can be dried blood spots (DBS), which minimize the cost of sample storage and transport. In this study, we developed a UPLC-MS/MS method for the analysis of rivaroxaban in DBS and plasma samples. Chromatographic separation was achieved on a Zorbax Eclipse Plus C18 column (2.1 × 100 mm; 3.5 µm, Agilent Technologies Inc., Santa Clara, CA, USA) with a mobile phase consisting of water and acetonitrile, both containing 0.1% formic acid. The analytes were detected using a positive ionization mode by multiple reaction monitoring. We validated the method according to ICH guidelines. The precision and accuracy were satisfactory. Extraction recovery was approximately 57% and 66% for DBS and plasma samples, respectively. A high correlation between rivaroxaban concentrations in plasma and DBS samples collected from patients was confirmed with Deming regression. The suitability of both sampling techniques for the rivaroxaban TDM was also verified by Bland–Altman plots based on DBS-predicted and observed plasma concentrations. In addition, we found a significant relationship between rivaroxaban concentrations and coagulation parameters, including prothrombin time (PT) and international normalized ratio (INR).

## 1. Introduction

Novel oral anticoagulants (NOAC) are currently more and more favored in anticoagulant therapy, due to their high safety and efficiency compared to vitamin K antagonists (VKA) [1,2]. Rivaroxaban, being part of the NOAC subgroup, is a direct inhibitor of Xa factor. Rivaroxaban is indicated for the prevention of venous thromboembolic events (VTE), the prevention of stroke and systemic embolism in patients with nonvalvular atrial fibrillation, and the treatment of deep vein thrombosis (DVT) and pulmonary embolism [2,3]. Rivaroxaban, compared to VKA, is characterized by its high safety profile, good bioavailability, and rapid onset, and it does not require routine monitoring of blood coagulation parameters. Despite this higher safety profile, rivaroxaban therapy is still connected with such multiple adverse effects as a higher risk of hemorrhage and bleeding (e.g., gastrointestinal hemorrhage, nose and gum bleeding), hematuria, or Stevens–Johnson syndrome (most often at the beginning of the therapy) [4,5]. On the other hand, several reports describe cases of non-responders who experienced recurrent thromboses while receiving rivaroxaban [6,7,8,9]. Foregoing events can endanger patients’ wellbeing and possibly impair the efficacy and safety of rivaroxaban therapy.

The bioavailability of the drug can be altered by administration with food, and higher absorption is observed after meal intake. Better bioavailability (39% higher mean AUC) after administration of 15 mg and 20 mg of rivaroxaban with a meal was observed in comparison to lower doses (2.5 and 10 mg) [10].

The majority of the rivaroxaban dose is metabolized by CYP3A4 and CYP2J2 enzymes. The effectiveness of rivaroxaban therapy could possibly be influenced by inhibitors (e.g., clarithromycin, diltiazem, ketoconazole, ritonavir, verapamil, and grapefruit) or inducers (e.g., phenytoin, phenobarbital, St, John’s Wort, and glucocorticoids) of CYP enzymes [11,12]. Additionally, rivaroxaban is a substrate for breast cancer resistance protein (BCRP) and P-glycoprotein (ABCB1) engaged in membrane transport, and the presence of other substrates (imatinib, rosuvastatin, methotrexate, pantoprazole) may lead to competitive displacement for binding sites resulting in altered absorption and elimination processes [12,13]. Thus, interactions with food, herbal supplements, and drugs should be considered in patients treated with rivaroxaban as they may affect its plasma concentrations [14]. The influence of gene polymorphisms of CYP3A4, CYP2J2, BCRP, and ABCB1 may affect the protein functionality and alter the response to rivaroxaban therapy [12,15,16,17].

The aforementioned factors lead to high interindividual variability (e.g., systemic clearance with a percent coefficient of variation ranging 30–40%) [10]. With this in mind, a prediction of the mentioned side effects is not easy. Moreover, adjusting the appropriate therapeutic dose and ensuring the safety and efficacy of rivaroxaban therapy might prove to be a laborious task. There are clinical conditions where accurate knowledge of drug concentrations may be necessary for the management of the anticoagulant therapy (e.g., liver or kidney dysfunction, bleeding breakthrough) [18]. Therapeutic drug monitoring (TDM) might also be useful in patients with BMI > 40 kg/m^2^ or those who are underweight (<50 kg) and are therefore at risk of not reaching therapeutic concentrations of NOAC [19].

Indirect methods for the measurement of rivaroxaban pharmacodynamic effect include chromogenic anti-Xa assays, prothrombin time (PT) tests, and activated partial thromboplastin time (aPTT) tests [20]. PT and aPTT measurements are characterized by low sensitivity and specificity, which makes them hard to utilize while assessing the efficacy of the therapy. Anti-Xa assays are postulated to be suitable for the determination of rivaroxaban. However, they measure inhibition of factor Xa, which is not specific to the NOAC [21]. Thus, TDM of rivaroxaban based on measurements of its concentrations was suggested [17,22]. Currently, the HPLC-MS/MS method is a gold standard for measuring rivaroxaban plasma/serum concentrations due to its high specificity, sensitivity, and reproducibility [23,24,25]. There is no strictly established therapeutic window for rivaroxaban concentrations. Based on recommendations of the International Council for Standardization in Hematology, minimal and maximal concentrations of rivaroxaban should range 12–137 ng/mL and 184–343 ng/mL, respectively [26].

An alternative for plasma samples can be dried blood spots (DBS), which minimize the costs of sample storage and transport. DBS samples are widely used for screening, genetic, and metabolite analysis [27,28,29,30]. DBS-based methods for application in TDM are still being developed [28,29,31,32,33,34]. Currently, this sampling technique can be used for the measurement of blood concentrations for antidepressants (e.g., amitriptyline), antibiotics (e.g., vancomycin), antiepileptic drugs (e.g., phenytoin), and narcotic substances (e.g., opioids) [32,35,36,37]. Samples of venous and capillary blood stored as DBS are generally more stable and take up less space than venous or plasma samples. Thus, they are more convenient for long-term storage [38,39,40].

However, in the case of DBS samples, there are more factors that could impact the outcome of analyses and the final results observed. This is why, during an analytical method validation, it is necessary to include the evaluation of additional parameters as recommended by the FDA [41]. For necessary reproducibility, the sample must create an area large enough so that the punching can be applied. Moreover, the analyte must be distributed equally throughout the blood spot. High hematocrit levels are often parallel to higher viscosity of blood, which could, therefore, impact how fast blood will spread on the DBS card. Hence, hematocrit levels could possibly influence the concentration levels of the analyte in DBS samples, and its impact should be estimated [42,43].

In this study, we developed and validated the UPLC-MS/MS method as useful for the analysis of rivaroxaban in DBS and plasma samples. The DBS technique requiring the collection of 20 μL blood proved to be an attractive low-blood-volume alternative to traditional venipuncture sampling. The advantages of the developed UPLC-MS/MS method were a fast and straightforward sample preparation (protein precipitation) and a short analysis time of 2 min. The novelty is a lower LLOQ than previously reported for DBS and detailed results of the method validation in both matrices, plasma and DBS, in contrast to other studies focusing only on DBS or plasma/serum (Table S1). In addition, we presented an analysis of the correlation between rivaroxaban concentrations in DBS and plasma and coagulation parameters, including blood levels of d-dimers which are still poorly studied. The application of the method was confirmed in the analysis of clinical samples obtained from patients with venous thrombosis. The determined concentrations in both DBS and plasma were correlated with coagulation parameters and proved to be useful in monitoring the effectiveness of anticoagulant therapy.

## 2. Results

### 2.1. UPLC-MS/MS Conditions

MS conditions were adjusted for the highest possible sensitivity to rivaroxaban and rivaroxaban-d4. The column and mobile phase composition were chosen for good resolution of peaks for each compound. Figure S1 shows chromatograms acquired for a blank sample, a sample spiked with rivaroxaban at a lower limit of quantification (LLOQ), and a sample obtained from a patient with venous thrombosis treated with 20 mg of rivaroxaban (for both DBS and plasma samples).

### 2.2. Method Validation

#### 2.2.1. Selectivity

Selectivity was determined by analyzing six blank DBS and plasma samples obtained from different sources. The results indicated that no interference from the matrix was present during the analysis.

#### 2.2.2. Calibration Curve

Calibration curves were analyzed at nine-point levels for rivaroxaban. The concentration range of 2–500 ng/mL was established based on expected plasma concentrations in patients under a standard dose regimen.

The calibration curve was linear in the range of 2–500 ng/mL for both DBS and plasma samples, and the correlation coefficient was ≥0.997 (Figures S2 and S3).

#### 2.2.3. LLOQ, Accuracy and Precision

The LLOQ was estimated at the lowest calibration standard concentration of 2 ng/mL, where the relative standard deviation (RSD%) and the relative error (RE%) were 10.43–14.21% and 4.77–10.05% for plasma samples and 3.85–10.89% and 9.71–16.55% for DBS, respectively.

The precision and accuracy for the analyte prepared at low, medium, and high levels in six replicates each were in the ranges of 1.70–14.54% and 0.91–12.79%, respectively. The results were in accordance with ICH M10 guidelines. The results, both for DBS and plasma samples, are shown in Table 1.

#### 2.2.4. Recovery and Matrix Effect

Extraction recovery of rivaroxaban from DBS samples was lower than from plasma samples (56.56 ± 8.58% versus 67.03 ± 6.02%, respectively). The results are displayed in Table 2.

We investigated the influence of matrix components on the ionization of rivaroxaban at concentrations of 5 and 500 ng/mL. Matrix components did not significantly suppress or enhance the MS signal of the analyte, as proved by the IS-normalized matrix factor (MF) ranging from 0.96–1.04 for plasma and 0.93–0.97 for DBS. The RSD% of the measurements was <15%.

#### 2.2.5. Dilution Integrity

A dilution factor of two did not influence the analysis results. Both accuracy and precision were within 15% bias (RE%—2.64% and RSD%—4.81%, respectively).

#### 2.2.6. Stability

The rivaroxaban in DBS samples stored at +4, −25, and −80 °C was stable for at least 113 days (Table 3). Long-term stability testing proved that rivaroxaban is stable in plasma samples stored at −25 °C for at least one month (RE% ranged within 0.5–3.06%), as shown in Table 4. The rivaroxaban isolated from plasma was also stable in samples stored in the autosampler for at least 24 h. The samples containing the analyte isolated from DBS were stable when stored in the autosampler for at least 48 h (Table 3). Regarding top-bench stability, rivaroxaban in both plasma and DBS samples was proven to be stable for at least 4 h, as shown in Table 3 and Table 4. Working solutions were stable for at least 297 days at −25 °C (RE% equal 3.85 and 11.04 for 2 ng/mL and 500 ng/mL, respectively).

#### 2.2.7. Correlation of the Rivaroxaban Concentration in DBS and Plasma

Concentrations of rivaroxaban in DBS and plasma samples obtained from drug-spiked blood were compared by regression analysis, which demonstrated that DBS and plasma concentrations were highly correlated (R^2^ = 0.9961) (Figure S4).

#### 2.2.8. Hematocrit and Blood Spot Volume

We did not observe the influence of hematocrit on the results of rivaroxaban analysis at concentrations of 5 ng/mL (RSD% 1.55–2.74%; RE% 0.07–2.53%) and 500 ng/mL (RSD% 3.20–6.47%; RE% 0.03–2.75%). We did not observe the influence of sampling spot volume, as both precision and accuracy were within 15% bias. The results are displayed in Table 5.

#### 2.2.9. Clinical Samples

**Figure 1:**
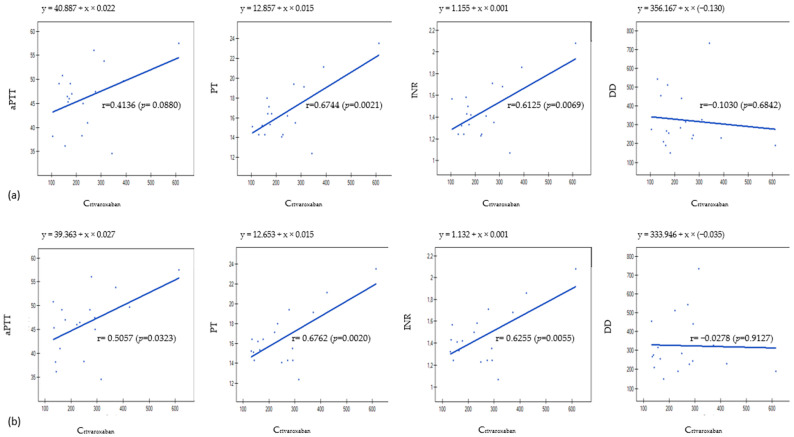
Results obtained from Pearson’s correlation analysis for (**a**) DBS samples and (**b**) plasma samples. aPTT—activated partial thromboplastin time, PT—prothrombin time, INR—international normalized ratio, DD—d-dimers.

The validated UPLC-MS/MS method was successfully applied for the measurement of rivaroxaban concentrations in plasma and DBS samples collected from 18 patients with venous thrombosis. The observed concentrations were within the ranges of 104.64–611.63 ng/mL in DBS and 131.00–614.32 ng/mL in plasma samples. In samples obtained from three patients, the concentrations exceeded the values recommended by the International Council for Standardization in Hematology [26] and may increase the risk of bleeding. We performed a simple correlation assessment between concentrations of rivaroxaban and coagulation parameters based on the Pearson correlation coefficient. We found a significant relationship between rivaroxaban concentrations in DBS and PT and international normalized ratio (INR) (r = 0.6744, *p* = 0.0021 and r = 0.6125, *p* = 0.0069, respectively). In the case of plasma samples, we found a correlation between rivaroxaban concentrations and aPTT (r = 0.506, *p* = 0.0323), PT (r = 0.676, *p* = 0.0021), and INR (r = 0.626, *p* = 0.0055) parameters. Blood levels of d-dimers (DD) did not correlate with rivaroxaban concentrations in DBS and plasma (*p* > 0.05). The results are displayed in Figure 1.

**Figure 2:**
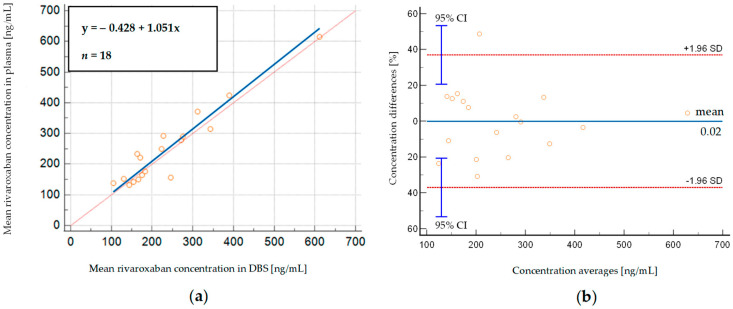
(**a**) Deming regression of rivaroxaban concentrations measured in DBS whole-blood samples, displayed against simultaneously analyzed plasma samples obtained from patients; (**b**) Bland–Altman plot, depicting % differences between DBS—predicted and measured plasma rivaroxaban concentrations. SD—standard deviation, 95% CI—95% confidence interval.

In order to check if the concentrations of rivaroxaban in DBS correspond to those determined in plasma samples, we used Deming regression. The slope of Deming regression of 1.05 (95% CI: 0.82–1.28) suggests that plasma concentrations correspond to the respective DBS concentrations (Figure 2a). The high regression coefficient (R^2^ = 0.950) indicates that more than 95% of rivaroxaban concentrations observed in plasma can be estimated based on rivaroxaban concentrations determined in DBS samples.

We then compared the concentrations measured in 18 plasma samples with those calculated from the corresponding DBS samples using Bland–Altman analysis. Figure 2b shows that there is a relatively small mean% difference between predicted and observed rivaroxaban concentrations (−0.02%), considering the high rivaroxaban concentrations observed in patients (131.00–614.32 ng/mL and 104.64–611.63 ng/mL in plasma and DBS, respectively). Most measurements (94.44%) lie within ±1.96 SD (limits of agreement) of the mean difference, which is close to the recommended 95% value, and there is only one outlier [44]. The obtained results suggest that both sampling methods provide similar results, and DBS and plasma samples can be used interchangeably for monitoring rivaroxaban concentrations.

## 3. Discussion

NOACs, due to their reliability, safety, and high efficacy, are more and more frequently used in anticoagulant therapy [1,2]. However, despite a higher safety profile compared to warfarin, rivaroxaban therapy is still connected with multiple adverse effects [4,5]. At the same time, several reports describe cases of non-responders who experienced recurrent thromboses while receiving rivaroxaban [6]. The abovementioned reasons caused an increase in interest in TDM of rivaroxaban. However, whole-venous blood sample collection is a demanding task as it requires qualified medical staff and strict storage conditions. Currently, new matrices and new sampling techniques are being searched for to overcome these challenges. DBS samples are much easier to collect, and in the case of rivaroxaban, they are proven to be, in many cases, much more stable than respective whole blood samples [38]. In that case, it could be beneficial to store already collected whole blood samples as DBS, which would enhance sample stability and reduce the costs and possible disadvantages of sample storage. In the present study, the applied DBS sampling technique requiring the collection of 20 μL whole blood proved to be an attractive alternative to traditional venipuncture sampling.

For analysis of rivaroxaban in DBS and plasma, a fast and simple UPLC-MS/MS method was developed and validated based on ICH guidance [45] regarding selectivity, linearity and LLOQ, precision and accuracy, matrix effect, carry-over, and stability under different conditions. The calibration curve was linear in the concentration range of 2–500 ng/mL for both matrices. Additionally, we assessed the influence of dilution, as we observed that there were patients’ samples with analyte concentrations beyond the calibration curve range. Dilution did not affect the accuracy of the results, which indicates that this method is suitable for the analysis of rivaroxaban concentrations up to 1000 ng/mL in clinical samples. Established LLOQ was 2 ng/mL, which was lower for DBS compared to other reported methods (2.06 ng/mL [46], 2.5 ng/mL [31], and 10 ng/mL [38]) (Table S1). Retention time for both rivaroxaban and IS was approximately 2 min and was relatively short, compared to methods described by Jahangir et al. [47] (approximately 3 min) and Brückner et al. [25] (5.45 min).

Acquired intra-day RSD% and RE% for DBS samples were 1.87–7.66% and 1.16–16.55%, respectively. The obtained intra-day precision was better than that reported by Foerster et al. [31] (1.1–13.0%) and Iqbal et al. [46] (3.03–10.0%). However, we obtained less intra-day accuracy compared to Jhang et al. [38] (99.8–104.3%) and Iqbal et al. [46] (92.0–112.7%). Regarding inter-day precision and accuracy, RSD% and RE% equaled 1.70–12.09% and 1.26–9.71, respectively. Our RE% results were similar to those reported by Foerster et al. [31] (92.0–94.0%) and Jhang et al. [38] (95.2–104.1%). Concurrently, we achieved a smaller accuracy range than Iqbal et al. [46] (92.4–113.6%).

Regarding plasma samples, the achieved intra-day RSD% was 7.86–14.54%, which was similar to results reported by Derogis et al. [24] (1.3–12.9%); however, the authors obtained better inter-day precision (3.7–8.8%, compared to 5.54–10.43%). The established inter-day RE% was within the 0.91–10.05% range and was close to results reported by Derogis et al. [24] (93.3–101.6%). Regardless of the abovementioned differences, our results were within the 15% bias (20% for LLOQ) required by ICH M10 guidelines [45].

The extraction recovery of rivaroxaban from DBS samples was lower than from plasma samples (56.56 ± 8.58% and 67.03 ± 6.02%, respectively). We obtained higher values than Iqbal et al. [46] (43.9%), even though we used the same extraction solvent. Higher values of 75–88% were obtained by Foerster et al. [31], who used a multi-step procedure involving sonication and shaking. Similarly, Jhang et al. [38] isolated rivaroxaban with efficiency of 100%, using two-step extraction and tissue homogenizing.

We did not observe a matrix effect during validation for both plasma and DBS samples. These results are in accordance with previously published reports [31,46]. Different results obtained by Jhang et al. [38], as they observed the matrix effect, ranged between 22.5 and 86.8%.

To ensure the stability of rivaroxaban in the studied matrices, we performed a series of stability tests at different storage conditions. Our results indicate that rivaroxaban is stable in DBS for at least 3 months in all studied temperatures (4, −25 and −80 °C). Additionally, we evaluated the analyte stability in plasma samples and found that they can be stored for at least 1 month at −25 °C. Iqbal et al. [20] widened their research regarding stability in DBS to a high temperature of 40 °C and found that rivaroxaban is stable in these conditions for at least 3 days. However, they did not study the analyte’s stability in corresponding plasma samples. High stability of rivaroxaban was also confirmed by other authors in plasma and DBS samples [31,38]. In other studies, the DBS samples were proved to be more stable than whole-blood samples (for up to 2 h and up to 329 days at room temperature, for plasma and DBS, respectively) [31,48].

One of the obstacles to introducing DBS sample analysis in routine practice is additional factors, which have to be taken into consideration during the validation process. The hematocrit effect can directly impact blood viscosity and hence influence analyte distribution on DBS cards. Currently, new technologies are being developed in order to minimize the influence of hematocrit on DBS analysis, based on new DBS cards’ structure [34]. Moreover, it has been suggested that covering the whole designated spot during sample preparation could lower the hematocrit influence on obtained results [35]. We did not observe the influence of hematocrit and sampling spot volume on rivaroxaban concentrations, as both precision and accuracy were within a 15% bias.

The UPLC-MS/MS validated method was successfully applied for the measurement of rivaroxaban concentrations in plasma and DBS samples collected from patients with venous thrombosis. The observed concentrations were within ranges of 104.64–611.63 ng/mL in DBS and 131.00–614.32 ng/mL. Considering the fact that all patients were treated with 15 or 20 mg of rivaroxaban, such inter-individual variability of the observed concentrations and exceeding the recommended therapeutic window might be an indication for the introduction of TDM to prevent bleeding, as has been suggested by other authors [23,49,50].

We established the correlation of rivaroxaban concentration between DBS and plasma using the Deming regression, which suggests that plasma concentrations are similar to respective DBS concentrations, as the slope of the curve equals 1.05. Our result corresponds to those previously published [31,38]. However, we observed less disparity than was reported by Jhang et al. [38] and Foerster et al. [31] (the slopes of Deming regression equal 1.3 and 1.37, respectively). At the same time, Iqbal et al. [46] reported lower rivaroxaban concentrations in plasma samples compared to respective DBS samples, but their analysis was based on four clinical samples only. The high regression coefficient (R^2^ = 0.950) indicates that more than 95% of rivaroxaban concentrations observed in plasma can be estimated based on rivaroxaban concentrations determined in whole-blood DBS samples.

As the routine coagulation screening test remains the most readily available method to assess DOAC anticoagulant activity, we checked if PT, aPTT, INR, and DD are related to the determined rivaroxaban concentrations. PT and INR showed a moderate correlation with rivaroxaban levels in DBS and plasma, while for aPTT, a moderate correlation was observed only for plasma samples. Hence it might be challenging to make therapeutic decisions based on these blood parameters only. Similar conclusions were reached by Kaserer et al. [20]. The authors found a moderate correlation of PT, INR, and aPTT with rivaroxaban plasma concentrations. However, in many samples, despite normal values of PT (50% of all samples), INR (25% of all samples), and aPTT (80% of all samples), residual rivaroxaban plasma concentrations were high (>50 ng/mL) and prohibitive for surgery. Regardless of the found correlation, the authors stated that standard coagulation assays are not sufficient for detecting relevant rivaroxaban plasma concentrations. The same conclusion was reached by He et al. [51]. They found a moderate correlation between rivaroxaban concentrations in plasma and PT/INR parameters. However, routine coagulation assays (PT, INR, aPTT, and thrombin time—TT) could not detect trough rivaroxaban concentrations below 50 ng/mL. They also stated that aPTT and TT assays were insufficient for predicting rivaroxaban trough concentration at any level. Routine coagulation assays are insufficient to monitor therapy with rivaroxaban, as the prolongation of PT, INR, or aPTT may be due to other factors, such as compromised liver function, antibiotic use, or vitamin K deficiency; hence, they might lead to false conclusions about rivaroxaban effectiveness resulting in a higher risk of adverse effects or impairment of the treatment [20,51]. This is why measurement of rivaroxaban concentrations might be beneficial for patients’ safety and efficient anticoagulant treatment. Baturina et al. suggested a similar approach in their work [17]. The authors stated that a higher concentration of rivaroxaban (Css min > 137 ng/mL), along with genetic analysis of ABCB1 rs4148738 polymorphism, could be beneficial for high-risk patients during antithrombotic treatment.

## 4. Materials and Methods

### 4.1. Chemicals and Reagents

Reference standards of rivaroxaban (99.76% purity) and rivaroxaban-d4 (97% purity) were purchased from LGC Standards Ltd. (Kiełpin, Poland). LC-MS grade methanol and acetonitrile were obtained from J.T. Baker (Phillipsburg, NJ, USA), while LC-MS grade water was from Witko Ltd. (Łódź, Poland). Formic acid (98–99% purity) was supplied by Merck (Darmstadt, Germany). Blank human plasma and whole blood of individual donors for method validation were received from the Center of Blood Donation (Poznań, Poland).

### 4.2. UHPLC-MS/MS and Chromatographic Conditions

We performed analysis using a Shimadzu UPLC Nexera set (Shimadzu Co., Kyoto, Japan) equipped with a five-channel degasser (DGU-20A5) and thermostated autosampler (SIL-30AC). The UPLC set was connected to a triple-quadrupole mass spectrometer (LCMS-8030). For data processing, the LabSolutions Series Workstation system (Shimadzu, Kyoto, Japan) was used.

We used the Zorbax Eclipse Plus C18 column (2.1 × 100 mm; 3.5 µm; Agilent Technologies Inc., Santa Clara, CA, USA) for chromatographic separation. The mobile phase consisted of water and acetonitrile (1:1, *v*/*v*), both containing 0.1% formic acid (*v*/*v*). Mobile phase flow was 0.2 mL/min. The column was kept at 40 °C temperature (CTO-2AC column oven, Shimadzu). The volume of injected sample was 5 µL. Retention time for both rivaroxaban and rivaroxaban-d4 (internal standard, IS) was approximately 2 min. During the method development, we evaluated several analytical columns and mobile phase compositions with different organic solvents (acetonitrile, methanol) and additives (formic acid, ammonium formate) to obtain the best peak shape of the analyte in a reasonable time. Other tested columns were Kinetex C18 (100 × 2.1 mm, 2.6 µm, Phenomenex, Torrance, CA, USA), Kinetex F5 (100 × 2.1 mm, 2.6 µm, Phenomenex), and Zorbax SB-C8 (150 × 4.6 mm, 5 μm, Agilent Technologies Inc.).

MS/MS detection was accomplished in positive electrospray ionization (ESI+) and multiple reaction monitoring (MRM) modes. We used nitrogen as a nebulizing gas (2 L/min) and drying gas (10 L/min). Electrospray voltage equaled 4.5 kV. The heat block temperature was adjusted at 400 °C. We used a water and acetonitrile mixture (1:1, *v*/*v*) to minimize the carry-over effect. The highest transition intensity was observed from *m*/*z* 435.90 to 144.85 for rivaroxaban, and from *m*/*z* 440.10 to 144.90 for IS.

### 4.3. Standard Solutions

We prepared stock solutions by separately dissolving rivaroxaban and IS in methanol, obtaining 100 µg/mL concentration for both compounds. Then, we prepared working solutions of rivaroxaban by diluting the stock solution with methanol, obtaining the following concentrations: 20, 50, 100, 200, 500, 1000, 2000, 4000, and 5000 ng/mL. We prepared an IS working solution at a concentration of 100 ng/mL by diluting the rivaroxaban-d4 stock solution with methanol. The stock solutions were then stored at −80 °C, and the working solutions were stored at −25 °C. Additionally, we prepared a 10,000 ng/mL working solution from stock solution for the dilution integrity test.

#### 4.3.1. Preparation of Calibrators and Quality Control Samples

Samples for the DBS calibration curve were prepared by spiking 2 µL of respective working solutions into 18 µL of whole blood. We obtained the following calibration curve concentrations: 2, 5, 10, 20, 50, 100, 200, 400, and 500 ng/mL. Then, 20 µL of obtained sample was placed on a DBS card and left to dry for 2 h. We applied a puncher to cut out 6 mm circles from DBS samples and placed them into Eppendorf tubes. We added 20 µL of IS, 15 µL of 5% formic acid, and 280 µL of acetonitrile, and vortexed for 10 s. The next step was extraction in an ultrasonic bath for 25 min at 40 °C. After the extraction process, we transferred liquid into a fresh batch of Eppendorf tubes and evaporated samples for 30 min at 45 °C. Dry residue was reconstituted in 100 µL of acetonitrile and injected for HPLC-MS/MS analysis.

Samples for the plasma calibration curve were prepared by spiking 10 µL of respective working solutions into 90 µL of human plasma. We obtained the following calibration curve concentrations: 2, 5, 10, 20, 50, 100, 200, 400, and 500 ng/mL. Then, we added 10 µL of IS working solution. The sample was vortexed for 30 s. The next step was adding 15 µL of 50% formic acid and vortexing for 30 s. For protein precipitation, 400 µL of acetonitrile was used and vortexed for 1 min. After that, we centrifuged samples for 10 min (10,000× *g*) and transferred the supernatant to a new batch of Eppendorf tubes. Samples were then evaporated (90 min, 45 °C), and the dry residue was dissolved in 200 µL of acetonitrile and then injected for UPLC-MS/MS analysis.

Quality control samples (QCs) were prepared for plasma and DBS at the LLOQ (2 ng/mL), LQC (5 ng/mL), MQC (200 ng/mL), and HQC (500 ng/mL).

### 4.4. Method Validation

The validation of both bioanalytical methods was performed according to the guidelines of the European Medicine Agency (ICH guidelines M10 on bioanalytical method validation and study sample analysis) [45]. Parameters specific to DBS were evaluated as recommended by the FDA [41].

#### 4.4.1. Selectivity

The selectivity test was performed by analyzing the chromatograms of blank DBS and plasma samples from six individual sources and comparing them to the sample containing IS and rivaroxaban at the LLOQ level to check the potential interferences at their retention times. The signal intensity of the analyte should be ≤20% of that at LLOQ level and ≤5% for IS.

#### 4.4.2. Calibration Curves

Calibration curves were created for the ratio of the peak area of the analyte to that of the IS as a function of the analyte concentration covering the range of 2–500 µg/mL for rivaroxaban in plasma and DBS. Each calibration curve was analyzed using linear and non-linear regression with an appropriate weighting factor.

#### 4.4.3. LLOQ, Accuracy and Precision

LLOQ was defined as the lowest concentration of rivaroxaban determined by the method with precision and accuracy not exceeding 20%. The LLOQ samples with plasma and DBS concentrations of 2 µg/mL were prepared and determined.

Intra- and inter-day accuracy and precision were evaluated by analyzing QCs at LLOQ, LQC, MQC, and HQC in five replicates on the same day and over six different days.

The precision was estimated as RSD% = (SD/C_measured_)×100%. Accuracy was calculated as the RE% from the formula: ((C_measured_ − C_nominal_)/C_nominal_)×100%. The RSD% and RE% values should be within ±15% for QCs and ±20% for the LLOQ.

#### 4.4.4. Carry-Over Effect

The carry-over effect was measured by injecting the blank sample immediately after the calibrator at the highest concentration, each at five replicates. The signal of the blank sample should not exceed 20% of the analyte signal at the LLOQ level and 5% of the response for the IS.

#### 4.4.5. Recovery

Extraction recovery of rivaroxaban from biological matrices was investigated at the analyte concentrations of 5, 200, and 500 ng/mL in DBS samples and 5, 200, and 400 ng/mL in plasma samples. To determine the recovery, we prepared two series of samples (series 1 and series 2) for each matrix. Series 1 consisted of DBS or plasma samples spiked with respective rivaroxaban standard solution and IS, while samples of series 2 were spiked with pure methanol and IS. Then, both series were processed as described in Section 4.3.1. After evaporation, the dry residue of series 1 was dissolved in pure acetonitrile. Dry residue of series 2 was reconstituted in analyte solution at the same concentration as in the series 1 samples. Recovery was estimated using the following equation: (C_Series 1_/C_Series 2_) × 100%.

#### 4.4.6. Matrix Effect

The matrix effect was investigated for both plasma and DBS samples. It was evaluated by preparing and analyzing three replicates of LQC and HQC from six different sources. For each individual matrix lot, accuracy should be within 15% of the nominal concentration, and precision should not be greater than 15%. Moreover, MF was calculated by dividing the peak measured in a blank matrix spiked with the analyte after the evaporation procedure by the peak area of the analytes at equivalent concentrations in the absence of the matrix. Moreover, the IS-normalized MF was calculated by dividing the analyte’s MF by the IS’s MF. The RSD% of the IS-normalized MF should not be greater than 15%.

#### 4.4.7. Dilution Integrity

We performed dilution integrity tests to assess whether the dilution procedure affects results obtained during sample analysis in case there are any clinical samples beyond the concentration range of the calibration curve.

We performed the test by preparing a 10,000 ng/mL working solution of rivaroxaban. We injected 2 µL of working solution into 18 µL of whole blood and then diluted the sample with 20 µL of whole blood to obtain a concentration of 500 ng/mL. Then, we processed the sample according to the procedure described in Section 4.3.1. We analyzed the samples in five replicates, comparing them to the freshly prepared calibration curve. The RE% of the concentration value determined in diluted samples should be within ±15% of the nominal concentration, and the RSD% should not exceed 15%.

#### 4.4.8. Stability

The stability of rivaroxaban in DBS and plasma samples was assessed for LQC and HQC in three replicates under different storage conditions. For long-term stability tests, the DBS samples were stored at 4, −25, and −80 °C for 113 days. To test long-term stability in plasma, LQC and HQC samples were prepared and stored at −25 °C for 31 days. For autosampler stability, the samples were injected and then stored at 10 °C for 24 and 48 h. We assessed top-bench stability for both plasma and DBS samples by storing them on a benchtop for 4 h at room temperature. We also estimated the stability of the analyte in whole blood left at room temperature for 4 h. Additionally, we investigated the freeze–thaw stability of plasma samples after three cycles of freezing at −25 °C and thawing at room temperature. In addition, the stability of stock solutions and working solutions was investigated after storage at −25 °C for 297 days. According to ICH guidelines, the stability was confirmed if the deviation from nominal concentration did not exceed 15%.

#### 4.4.9. Hematocrit and Blood Spot Volume

The impact of hematocrit on determining rivaroxaban concentrations in DBS was tested by comparing lab-prepared samples with differing hematocrits (48.5, 40.0, and 31.5%). Blank whole-blood samples with hematocrit of 48.5% and 31.5% were obtained from male subjects. Additionally, the sample with a hematocrit of 48.5% was diluted with plasma to make whole blood with a hematocrit of 40%. The blank blood at each hematocrit was then spiked with rivaroxaban solution to represent the LQC and HQC levels and spotted in triplicate onto the DBS cards. The effect of hematocrit was negligible if the RE% was within 15% of the nominal concentration.

We also investigated the influence of blood spot volume on the accuracy of the determined rivaroxaban concentrations. For this purpose, we spotted different volumes of whole blood (15, 20, and 25 µL) spiked with rivaroxaban at LQC and HQC levels in triplicate on the DBS cards. The effect of blood spot volume was not significant if the deviation of the rivaroxaban concentration determined in 15, 20, and 25 µL of whole blood were within 15% of the nominal concentration.

#### 4.4.10. Correlation of the Rivaroxaban Concentration in DBS and Plasma

Before analysis of clinical samples, we compared rivaroxaban concentrations determined in the DBS and plasma spiked with the drug. Aliquots of 50 µL of working solutions at rivaroxaban concentrations of 200 and 10,000 ng/mL were added to 950 µL of fresh blood. The obtained blood concentrations of the drug were 100 ng/mL and 500 ng/mL, respectively. Then, 20 µL of the blood sample was placed on a DBS card (three samples for each concentration) and processed according to the procedure described in Section 4.3.1. The remaining blood was centrifuged for 10 min (3500× *g*). We transferred the obtained plasma into Eppendorf tubes and processed it in three replicates according to the procedure described in Section 4.3.1. We correlated results from the analysis of the DBS and respective plasma samples using a linear regression.

#### 4.4.11. Clinical Application

The clinical application of the UPLC-MS/MS method was demonstrated using samples obtained from 18 patients (10 females and 8 males, 53.83 ± 15.61 years old) suffering from deep vein thrombosis. Patients were treated with 15 mg and 20 mg doses of rivaroxaban daily. Aside from rivaroxaban concentrations, PT, aPPT, INR, and DD parameters were measured in the patients’ samples. Patient characteristics are presented in Table 6. The research was conducted according to the Declaration of Helsinki. The study was approved by the Bioethical Committee at Poznan University of Medical Sciences (no. 12/21). We obtained written consent from each participant. Approximately 2 h after rivaroxaban administration, a blood sample (5 mL) was collected into a tube containing ethylenediaminetetraacetic acid (EDTA). For plasma separation, the blood samples were centrifuged at 3500× *g* for 10 min. Obtained plasma samples were stored at −25 °C. Prior to analysis, 90 µL of plasma was transferred into Eppendorf tubes. Then, 10 µL of methanol was added. The following steps were the same as for the plasma calibration curve (Section 4.3.1.).

DBS samples were prepared by pipetting 20 µL of the patient’s whole venous blood on a DBS card, which was then left to dry. The cards were stored at 4 °C in plastic bags with desiccants. Prior to analysis, a blood spot was cut out using a puncher with a 6 mm diameter and placed in Eppendorf tubes. Then, the extraction procedure was performed as described in Section 4.3.1.

We compared concentrations of the analyte observed in DBS and in the plasma in order to calculate the correcting factor for the estimation of plasma concentrations based on those observed in DBS samples. Additionally, Deming regression and Blant–Altman analysis were applied for statistical comparison of the rivaroxaban concentrations in plasma and DBS samples.

## 5. Conclusions

We developed and validated a novel UPLC-MS/MS method for the analysis of rivaroxaban concentrations in DBS and plasma samples. The validation results support the advantages of DBS sampling, such as high stability and simplicity of storage. We successfully applied the method for analysis of the clinical samples obtained from patients with venous thrombosis and identified the subjects at high risk of bleeding. A comparison of the drug concentrations determined in plasma and respective DBS samples showed that DBS can be used as an alternative to more traditional whole-blood venipuncture in rivaroxaban therapeutic drug monitoring.

## Figures and Tables

**Table 1:** Inter-day and intra-day precision and accuracy for both DBS and plasma samples. The results are shown for LLOQ, low (LQC, 5 ng/mL), medium (MQC, 200 ng/mL), and high (HQC, 500 ng/mL) concentrations.

QC Concentration		Plasma Samples		DBS Samples	
		Intra-Day (*n* = 5)	Inter-Day (*n* = 6)	Intra-Day (*n* = 5)	Inter-Day (*n* = 6)
2 ng/mL (LLOQ)	RSD%	14.21	10.43	3.85	10.89
	RE%	−4.77	−10.05	−16.55	−9.71
5 ng/mL (LQC)	RSD%	7.86	7.81	1.87	12.09
	RE%	−2.68	−1.60	1.16	5.74
200 ng/mL (MQC)	RSD%	14.54	7.60	3.26	3.78
	RE%	−12.79	−1.74	−3.03	1.89
500 ng/mL (HQC)	RSD%	9.82	5.54	7.66	1.70
	RE%	0.99	0.91	3.00	−1.26

**Table 2:** Recovery and matrix effect of rivaroxaban after extraction from DBS and plasma samples.

Concentration of Rivaroxaban [ng/mL]	Extraction Recovery (Mean Value (RSD%))	Concentration of Rivaroxaban [ng/mL]	IS-Normalized MF (Mean Value (RSD%))
Plasma samples	*n* = 5		*n* = 6
5	71.73 (12.27)	5	0.96 (1.53)
200	60.24 (9.97)		
400	69.11 (8.04)	500	1.04 (1.24)
DBS samples	*n* = 5		*n* = 6
5	66.77 (10.08)	5	0.93 (4.86)
200	50.19 (7.15)		
500	52.17 (7.94)	500	0.97 (4.67)

**Table 3:** Results of stability tests for DBS samples: A—long-term stability test; B—autosampler stability; C—benchtop stability, D—stability of the analyte in whole blood at room temperature.

DBS Samples						
Stability Test	Conditions	Nominal Concentration [ng/mL]	Initial Mean Concentration ± SD [ng/mL] before Stability Test	Mean Concentration ± SD [ng/mL] after Stability Test	RSD%	RE%
A	+4 °C (113 days)	5	4.92 ± 0.06	5.42 ± 0.38	6.92	8.42
		500	524.87 ± 21.81	553.90 ± 44.14	7.97	10.78
	−25 °C (113 days)	5	4.92 ± 0.06	5.29 ± 0.15	3.94	5.89
		500	524.87 ± 21.81	499.80 ± 27.33	5.47	0.04
	−80 °C (113 days)	5	4.92 ± 0.06	4.96 ± 0.20	2.80	0.87
		500	524.87 ± 21.81	493.21 ± 14.90	3.02	1.36
B	10 °C (48 h)	5	4.93 ± 0.40	4.93 ± 0.47	9.47	1.49
		500	516.39 ± 7.90	557.62 ± 10.74	1.93	11.52
C	20 °C (4 h)	5	5.33 ± 1.24	4.90 ± 0.99	7.95	0.02
		500	511.60 ± 33.65	542.27 ± 13.47	5.99	8.45
D	20 °C (4 h)	5	5.00 ± 0.37	5.37 ± 0.47	8.72	7.44
		500	541.79 ± 41.70	547.98 ± 19.11	3.49	9.60

**Table 4:** Results of stability tests for plasma samples: A—long-term stability test; B—autosampler stability; C—benchtop stability; D—freeze–thaw stability test (after third freeze–thaw cycle).

Plasma Samples						
Stability Test	Conditions	Nominal Concentration [ng/mL]	Initial Mean Concentration ± SD [ng/mL] before Stability Test	Mean Concentration ± SD [ng/mL] after Stability Test	RSD%	RE%
A	−25 °C (31 days)	5	5.10 ± 1.04	4.54 ± 1.11	3.06	0.50
		400	399.51 ± 44.93	429.81 ± 0.75	2.50	3.06
B	10 °C (24 h)	5	5.48 ± 1.14	4.74 ± 0.32	2.72	1.65
		400	388.57 ± 37.54	392.16 ± 37.87	4.03	2.05
C	20 °C (4 h)	5	5.60 ± 0.61	6.23 ± 1.26	0.86	3.26
		400	338.53 ± 32.09	360.76 ± 26.72	3.11	0.09
D	−25 °C (3 cycles)	5	4.87 ± 0.77	4.31 ± 0.46	10.67	13.8
		400	366.98 ± 18.72	349.82 ± 9.16	2.62	12.55

**Table 5:** The influence of the hematocrit values and sampling spot volume on results obtained from DBS samples analysis.

Hematocrit Value [%]	LQC		HQC	
	RSD%	RE%	RSD%	RE%
48.5	1.55	2.53	6.47	0.39
40.0	2.63	0.07	3.20	2.75
31.5	2.74	1.73	3.84	0.03
Sampling spot volume [µL]				
15	6.61	4.27	2.38	4.30
20	10.72	8.61	0.47	2.00
25	7.92	5.05	1.90	5.64

**Table 6:** Characteristics of the studied group.

Parameter Measured (*n* = 18)	Mean ± SD
Age [years]	53.83 ± 15.61
Body weight [kg]	80.94 ± 16.69
Height [cm]	169.71 ± 7.94
BMI [kg/m^2^]	27.90 ± 4.13
aPTT [s]	46.20 ± 6.54
PT [s]	16.54 ± 2.78
INR	1.46 ± 0.25
DD [ng/mL]	271 (227.25–413.25) ^1^

^1^ results shown as median with lower and upper quartile, as DD parameter was not normally distributed, according to the Kolmogorov–Smirnov test.

## Data Availability

The data that support the findings of this study are available from the corresponding author upon reasonable request.

## Supplementary Materials

The following supporting information can be downloaded at https://www.mdpi.com/article/10.3390/molecules29174140/s1. Figure S1: Representative MRM chromatograms for the analysis of rivaroxaban in plasma and DBS samples: (A) blank sample, (B) plasma and DBS samples spiked with rivaroxaban at LLOQ, (C) plasma and DBS samples of a patient with venous thrombosis treated with 20 mg of rivaroxaban (determined concentrations: 277 ng/mL in plasma and 271 ng/mL in DBS). Figure S2: Mean calibration curve (*n* = 6) for DBS samples. Figure S3: Mean calibration curve for plasma samples. Figure S4: Rivaroxaban concentrations measured in respective DBS and plasma samples, calculated according to the DBS and plasma calibration curves. Correlation of observed concentrations, depending on the matrix used, was performed during the initial validation process. Table S1: Comparison of the cited methods for determination of rivaroxaban concentrations in both DBS and plasma/serum samples.
