# Supplementary material for: Application of a Novel UPLC-MS/MS Method for Analysis of Rivaroxaban Concentrations in Dried Blood Spot and Plasma Samples Collected from Patients with Venous Thrombosis
Source: Molecules. 2024 Aug 31;29(17):4140. doi: 10.3390/molecules29174140 (PMC11397208; doi:10.3390/molecules29174140)
Supplement: Supplementary file 1 [file molecules-29-04140-s001.zip › molecules-3179999-supplementary.pdf]

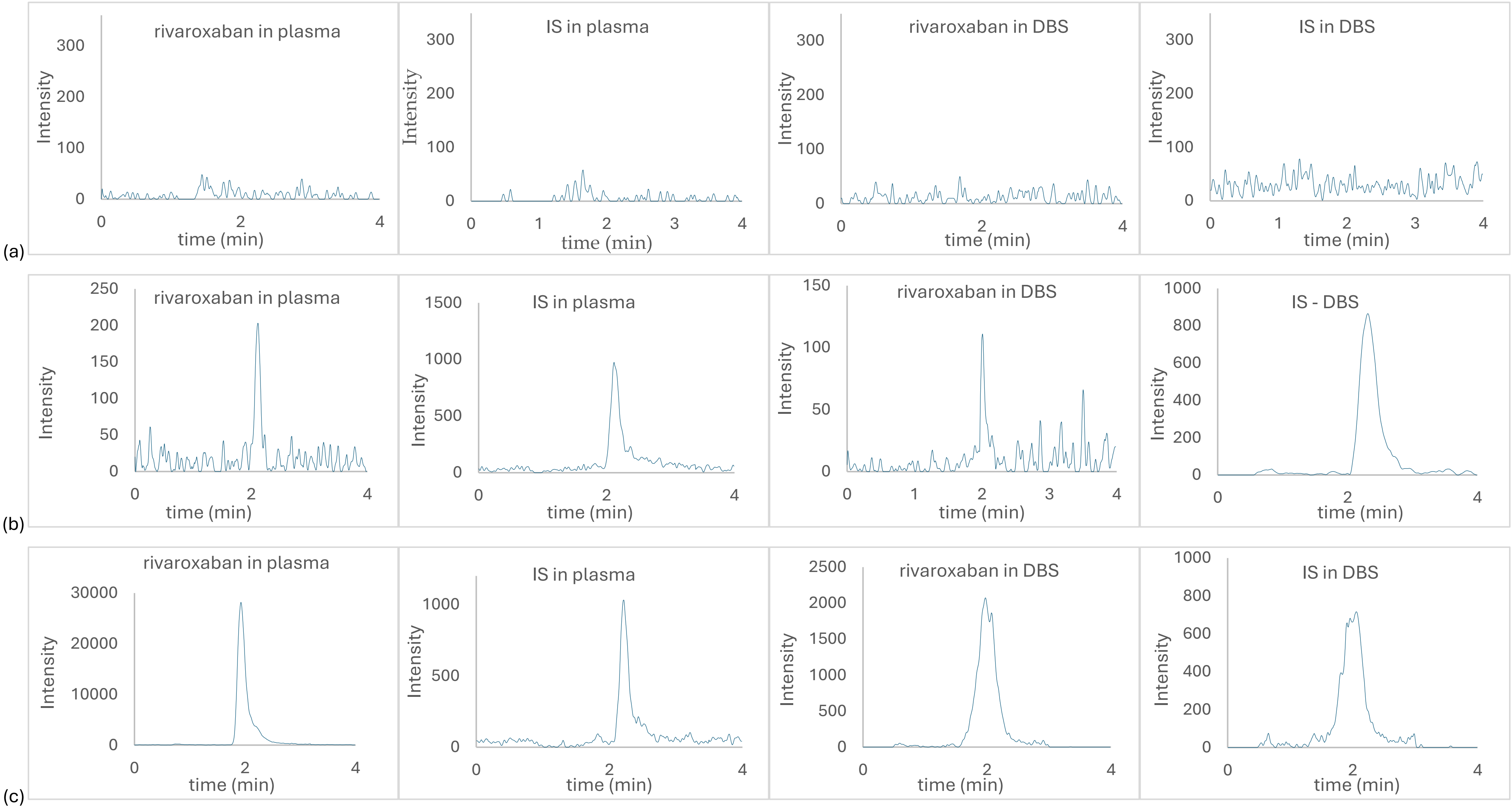

Figure S1. Representative MRM chromatograms for the analysis of rivaroxaban in plasma and DBS samples: (a) blank sample, (b) plasma and DBS samples spiked with rivaroxaban at LLOQ, (c) plasma and DBS samples of a patient with venous thrombosis treated with 20 mg of rivaroxaban (determined concentrations: 277 ng/mL in plasma and 271 ng/mL in DBS).

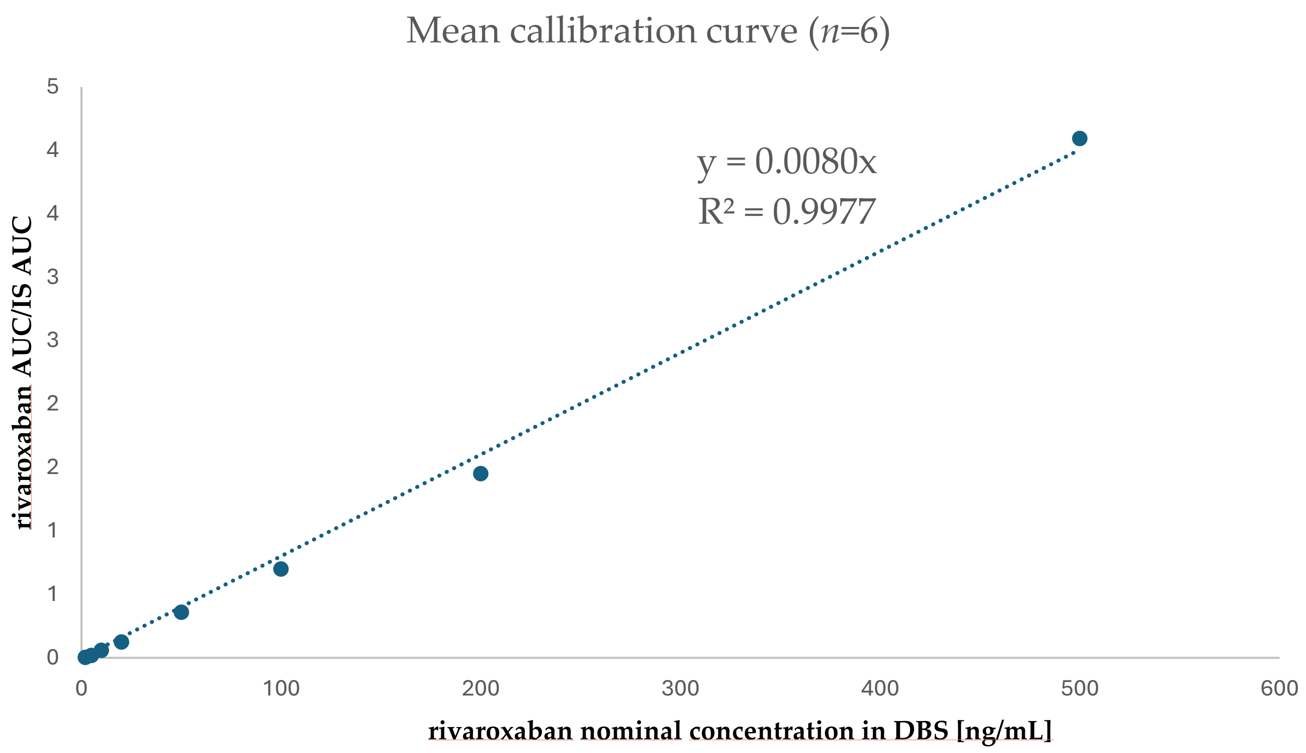

Figure S2. Mean calibration curve (n=6) for DBS samples.

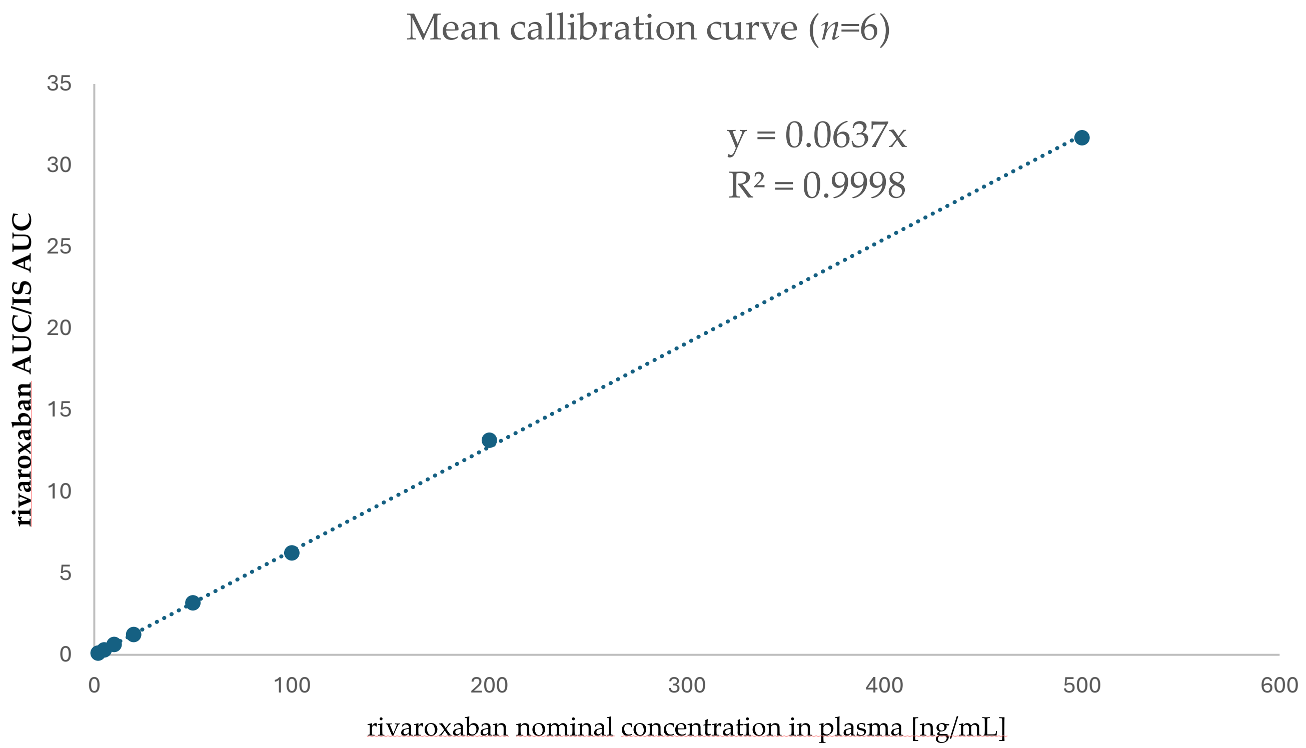

Figure S3. Mean calibration curve (n=6) for plasma samples.

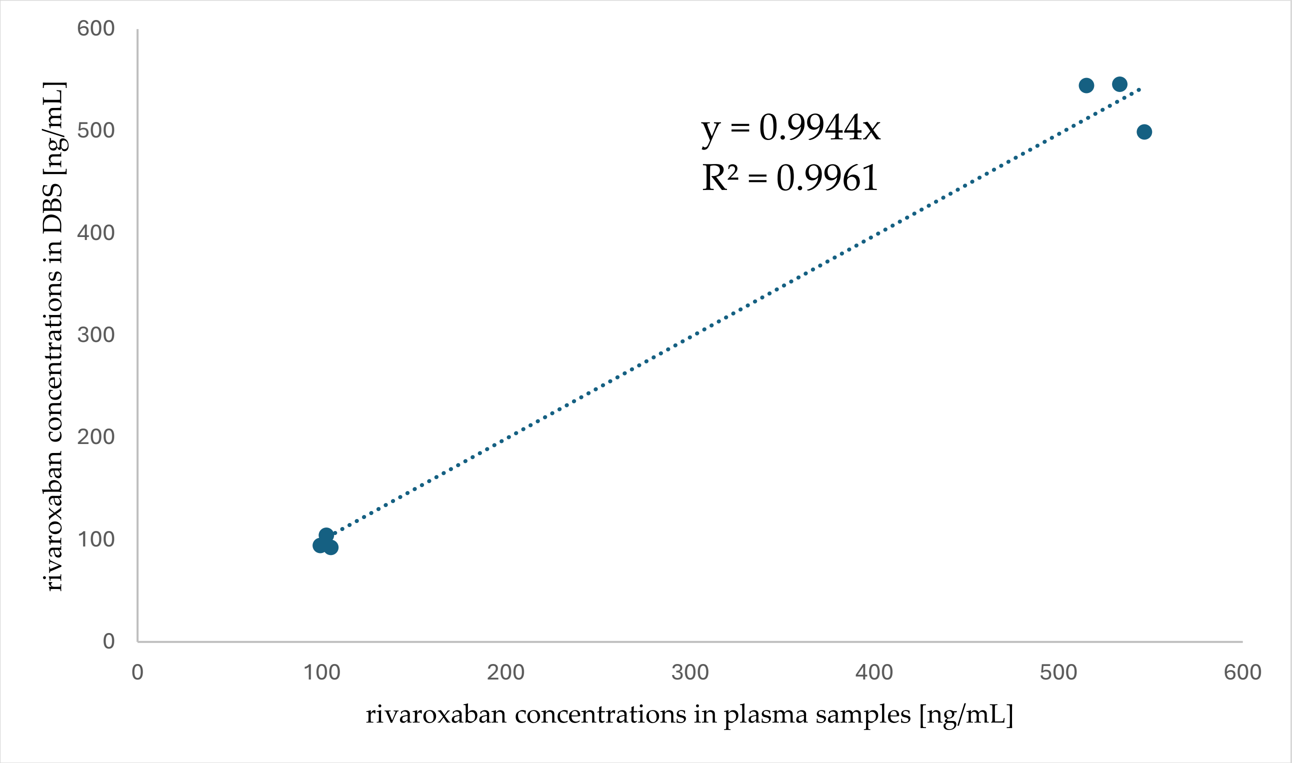

Figure S4. Rivaroxaban concentrations measured in respective DBS and plasma samples, calculated according to the DBS and plasma calibration curves. Correlation of observed concentrations, depending on matrix used, was performed during initial validation process.

Table S1. Comparison of the cited methods for determination of rivaroxaban concentrations in both DBS and plasma/serum samples.

n	Sample type	Analytical method	LOQ ng/mL	LOD ng/mL	Intra-day precision [RSD%]	Inter-day precision [RSD%]	Intra-day accuracy [%]	Inter-day accuracy [%]	Concentration range [ng/mL]	Recovery [%]	Reference
1	DBS	UPLC-MS/MS	2.5	NR	1.1-13.0	6.0-10.0	88.0-98.0	92.0-94.0	2.5-750.0	81.0	[31]
2	DBS	PC-IS LC-MS/MS	5	0.25	0.4-8.6	5.9-10.0	99.8-104.3	95.2-104.1	5–800	95.3-108.0	[38]
3	DBS	HILIC-based	2.06	NR	3.03-10.0	5.61-9.87	92.0-112.7	92.4-113.6	2.06-1000	43.9	[46]

		UHPLC-MS/MS								
4	plasma	RP-HPLC	670	220	1.78	1.76-1.93	98.5-102.9	NR	100000-400000	98.5-102.9	[47]
5	serum	HPLC–MS/MS	1.11	0.31	NR	10.7-14.9	NR	101.0-102.7	0.3–500	61.0-62.0	[25]
6	plasma	HPLC-MS/MS	2	NR	1.3-12.9	3.7-8.8	89.9-104.9	93.3-101.6	2-500	95.2-101.0	[24]
7	DBS	HPLC-MS/MS	2	1	1.87-7.66	1.70-12.09	97.00-116.55	94.26-109.71	2-500	50.19-66.77	Current method
	plasma		2	1	7.86-14.54	5.54-10.43	99.01-112.79	99.09-110.05	2-500	60.24-71.73	

NR - Not reported; accuracy was expressed as $(C_{measured}/C_{nominal})$*100%.
